# β-Cell Dysfunction, Hepatic Lipid Metabolism, and Cardiovascular Health in Type 2 Diabetes: New Directions of Research and Novel Therapeutic Strategies

**DOI:** 10.3390/biomedicines9020226

**Published:** 2021-02-23

**Authors:** Ahmad Al-Mrabeh

**Affiliations:** Faculty of Medical Sciences, Translational and Clinical Research Institute, Magnetic Resonance Centre, Newcastle University, Newcastle upon Tyne NE2 4HH, UK; ahmad.al-mrabeh2@ncl.ac.uk; Tel.: +44-(0)-191-2086768

**Keywords:** type 2 diabetes, lipoprotein metabolism, β-cell dysfunction, lipotoxicity, adipose tissue, weight loss, diabetes remission, cardiovascular disease, novel therapies

## Abstract

Cardiovascular disease (CVD) remains a major problem for people with type 2 diabetes mellitus (T2DM), and dyslipidemia is one of the main drivers for both metabolic diseases. In this review, the major pathophysiological and molecular mechanisms of β-cell dysfunction and recovery in T2DM are discussed in the context of abnormal hepatic lipid metabolism and cardiovascular health. (i) In normal health, continuous exposure of the pancreas to nutrient stimulus increases the demand on β-cells. In the long term, this will not only stress β-cells and decrease their insulin secretory capacity, but also will blunt the cellular response to insulin. (ii) At the pre-diabetes stage, β-cells compensate for insulin resistance through hypersecretion of insulin. This increases the metabolic burden on the stressed β-cells and changes hepatic lipoprotein metabolism and adipose tissue function. (iii) If this lipotoxic hyperinsulinemic environment is not removed, β-cells start to lose function, and CVD risk rises due to lower lipoprotein clearance. (iv) Once developed, T2DM can be reversed by weight loss, a process described recently as remission. However, the precise mechanism(s) by which calorie restriction causes normalization of lipoprotein metabolism and restores β-cell function are not fully established. Understanding the pathophysiological and molecular basis of β-cell failure and recovery during remission is critical to reduce β-cell burden and loss of function. The aim of this review is to highlight the link between lipoprotein export and lipid-driven β-cell dysfunction in T2DM and how this is related to cardiovascular health. A second aim is to understand the mechanisms of β-cell recovery after weight loss, and to explore new areas of research for developing more targeted future therapies to prevent T2DM and the associated CVD events.

## 1. Introduction

Type 2 diabetes mellitus (T2DM) has become a global concern. It affects 425 million of the world population, and doubling is predicted in the coming decades [1,2]. On average, 10–15% of the national health budget in western countries is directed to manage T2DM and its complications, including cardiovascular disease (CVD), the leading cause of death worldwide [3]. However, available drugs are relatively ineffective in controlling this epidemic, and there is urgent need for other means to manage T2DM and prevent development of CVD [4,5,6].

One of the major questions that remains a puzzle for people with T2DM and scientists alike is “why have I developed T2DM even though I’m not as heavy as my friends who do not have diabetes?” There are many factors that contribute to the pathophysiology of this disease, including body weight, fat mass, age, ethnicity, gender, genetics, and environmental factors [7]. However, the majority of these factors are modifiable and largely controlled by lifestyle intervention including diet and physical activities. Although it is considered a risk factor, obesity itself is not causative of T2DM [8,9]. It is known that the majority of overweight people do not develop T2DM [10]. This “metabolically healthy” phenotype is genetically determined via alleles associated with higher subcutaneous and lower ectopic fat deposition [11,12]. On the other hand, normal weight people may develop T2DM due to limited subcutaneous fat storage ability accompanied by susceptibility of insulin-producing cells within the pancreas (β-cells) to these adverse metabolic conditions, and the concept of personal fat threshold explains this phenomenon [9]. Subcutaneous adipose tissue allows metabolically well-tolerated fat storage, and this may partially explain why some people develop T2DM at normal body mass index (BMI) [9,10]. Indeed, women are generally less susceptible to T2DM and cardiovascular events, and large subcutaneous fat area in women may provide a safe zone for storage of excess triglycerides protecting the β-cells and other susceptible tissues from the harmful effects of excess lipids [7,13,14].

Genetic factors related to ability of β-cells to increase mass and function during high demand on insulin or ability of β-cells to survive the adverse metabolic conditions should be considered [15,16]. Work on animal studies support the beneficial effects of subcutaneous fat and the genetic basis of β-cell susceptibility to increased levels of glucose and fatty acids in obesity and T2DM [17,18]. In the lipodystrophy mouse model (A-ZIP/F-1), transplanting subcutaneous fat returned liver fat levels to normal and regulated blood glucose levels [19]. In addition, prefusion studies on isolated islets from the zucker diabetic fatty (ZDF) rat model have shown that simultaneous prefusion of fatty acids and glucose induced β-cell dysfunction in the homozygous rats, but not in the heterozygous littermates [18]. Genome-wide association studies (GWAS) have also highlighted the protective nature of subcutaneous fat in obesity and T2DM [12]. To date, the majority of identified polymorphisms in T2DM are related to the β-cell secretory function rather than insulin function itself [20,21]. However, it should be recognized that current studies identified less than 10% of the genetic factors that are expected to contribute to the pathophysiology of T2DM, and more genetic loci are expected to be identified in the future [15].

On the basis of recent evidence from the Diabetes Remission Clinical Trial (DiRECT), weight loss to treat T2DM is currently incorporated into the National Health System (NHS) in the United Kingdom, thereby to lower CVD risk [4,5]. However, achievement and maintenance of weight loss is difficult and requires strong motivation and long-term support for adherence to dietary conditions and prevention of weight regain following weight loss. In addition, this approach does not suit everybody, including those with normal body weight. There is a pressing need to develop more targeted strategies for remission of T2DM without the requirement for major weight loss.

**Figure 1:**
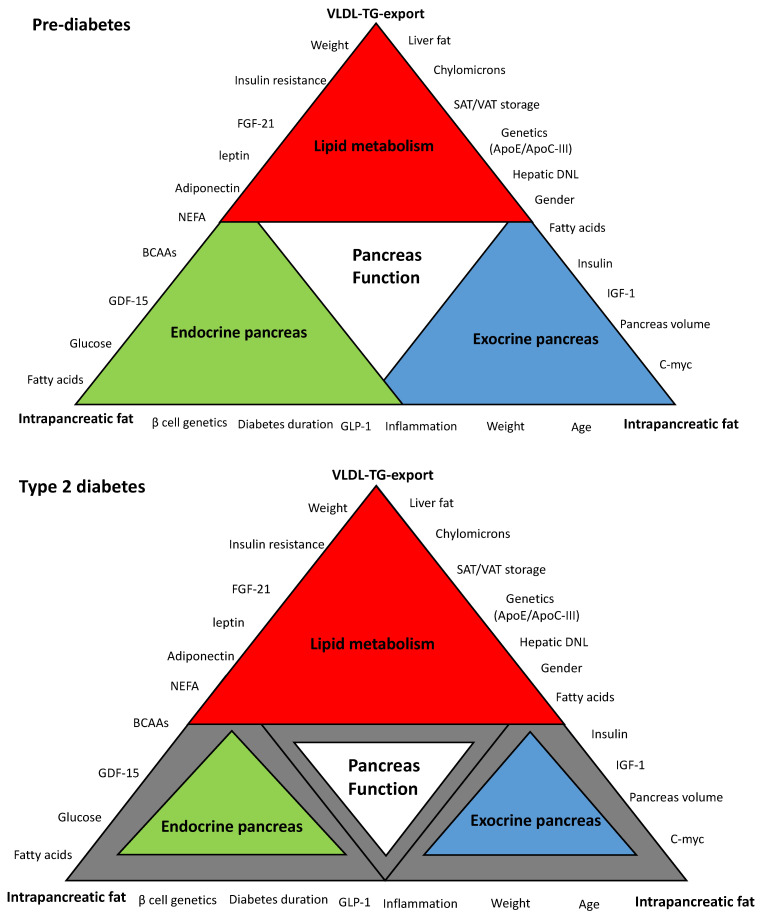
Schematic hypothetical diagram of the interplay between hepatic very low density lipoprotein triglyceride (VLDL-TG) export and intrapancreatic fat in type 2 diabetes. Pancreas function is accomplished through the synergy between endocrine and exocrine compartments. Abnormalities in lipid metabolism are the drivers of metabolic events that affect overall pancreas structure and function. The extent of each colored triangle represents the degree of function in that parameter (gray color means loss of function). Lipid-driven changes that cause dysfunction of β-cells in type 2 diabetes mellitus (T2DM) and the loss of the acinar cells mass are likely to be related to hepatic VLDL-TG export and increase in intrapancreatic fat. β-cell: Beta cell; VLDL-TG: very low density lipoprotein triglyceride; FGF-21: fibroblast growth factor 21; NEFA: non-esterified fatty acids; BCAAs: branched-chain amino acids; GDF-15: growth differentiation factor-15; GLP-1: glucagon-like peptide-*1*; *C-myc*: cellular myelocytomatosis oncogene; IFG-1: insulin-like growth factor-*1*; DNL: de novo lipogenesis; ApoE: apolipoprotein E, ApoC-III: apolipoprotein C-III; SAT: subcutaneous adipose tissue; VAT: visceral adipose tissue.

“Lipotoxicity” is one of the most widely accepted hypotheses to explain the underlying mechanisms of β-cell dysfunction in T2DM [16,22,23,24]. Maintenance of normal lipid homeostasis occurs via crosstalk among many organs of the body including the liver, the pancreas, and adipose tissues. Figure 1 is a schematic hypothetical representation of the lipid-related factors that may contribute to the pancreas function in T2DM. This review outlines and discusses the major pathophysiological and molecular mechanisms of β-cell dysfunction and recovery in T2DM in the context of dyslipidemia and cardiovascular health. Additionally, it will highlight new areas for future research to develop novel therapies for T2DM. Whether β-cell uptake of these toxic fatty acids and lipid intermediates delivered by lipoproteins could be blocked at early stages to preserve β-cells and maximize their survival requires investigation. Alternatively, novel therapeutic strategies are needed that enhance diversion/depletion of excess energy intake or healthy expansion of adipose tissues without the need to upregulation of the major lipogenesis and lipoprotein export pathways.

## 2. Altered Lipid Metabolism and Etiology of T2DM

Excess calorie intake and ectopic fat deposition are major determining factors for the pathophysiology of T2DM [25,26,27,28]. Obesity itself, defined as a fixed cut-off of body mass index (BMI), is not causative factor [8,9], and overweight people free from diabetes may have a “metabolically healthy” phenotype with safe fat storage [12,29]. On the other hand, those who develop T2DM at lower BMI may have (i) limited storage capacity or disordered adipose tissue function, (ii) varied susceptibility to toxic lipid metabolites, and (iii) failure to increase β-cell mass appropriately during adipose tissue expansion and increased demand on insulin [15,29,30].

In T2DM, lipid metabolism is abnormal, directly related to overproduction of very low density lipoprotein (VLDL) by the liver [31,32,33]. This is accelerated through expression of transcription factors that activate lipogenesis genes including the carbohydrate response element binding protein (ChREBP) and sterol regulatory element-binding protein 1c (SREBP1c), which are activated by glucose and insulin, respectively [34,35]. Therefore, the rates of hepatic de novo lipogenesis (DNL) rise substantially in T2DM under elevated levels of glucose and insulin [34,36,37].

**Figure 2:**
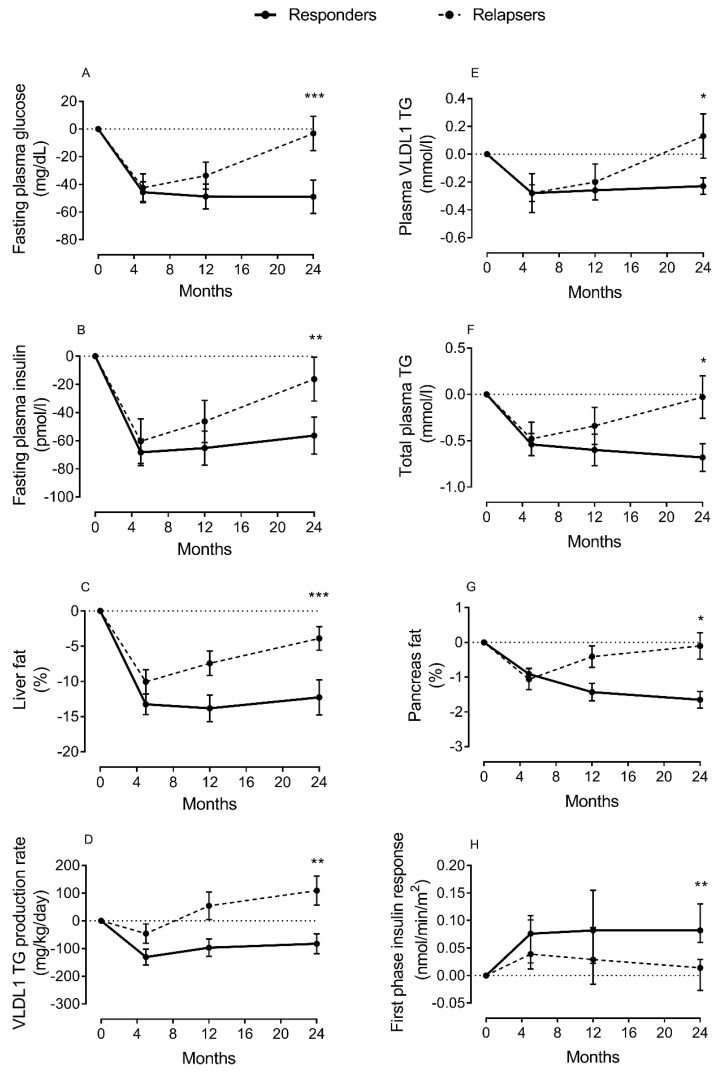
Change in lipid parameters and β-cell function during remission and relapse of type 2 diabetes. Change from baseline in fasting plasma glucose (**A**), fasting plasma insulin (**B**), liver fat (**C**), hepatic VLDL1-TG production (**D**), fasting plasma VLDL1-TG (**E**), total plasma triglycerides (TGs) (**F**), intrapancreatic fat (**G**), and β-cell function (**H**) at 5 months (responders *n* = 38; relapsers *n* = 13), 12 months (*n* = 28/*n* = 13, respectively), and 24 months (*n* = 20/*n* = 13, respectively). Responders are presented as a solid black line and relapsers as a dashed line. The dotted line is the gridline at *y* value = 0. Paired data between baseline and each time point are presented. Data are presented as mean ± SEM except for first-phase insulin (median with IQ range) vs. 5 months in relapsers: * *p* < 0.05, ** *p* < 0.01, *** *p* < 0.001. Figure is presented with permission [43].

Liver function is central for regulation of lipid metabolism through export of fat in lipoprotein (VLDL-TG), and uptake of free fatty acids from circulation [31]. However, this mechanism is impaired in T2DM [31,32]. As the liver fails to maintain the balance between fat uptake and delivery, excess fat will be stored ectopically when subcutaneous depots are unable to accommodate more triglycerides. Thus, non-alcoholic fatty liver disease (NAFLD) is common, and directly involved in the pathogenesis of T2DM [38,39,40,41]. Excess fat will not only impair liver function in regulating blood glucose levels, but will also spillover to other ectopic sites, including the pancreas and muscles, interfering with β-cell function and cellular insulin signaling. Using magnetic resonance imaging (MRI) and intralipid infusion techniques, we have reported elevated levels of liver fat and major increase in hepatic VLDL-TG production in T2DM, and that this was normalized after remission of diabetes [26,27,42] (Figure 2). If DNL is the driver of excess liver fat accumulation and hepatic VLDL-TG production, it would be expected to decrease during remission of T2DM, and this would possibly by a target for the future prevention and remission programs.

One other important function of the liver is uptake and clearance of lipoprotein remnants from the blood, which results from the catabolism of triglyceride-rich lipoproteins. This includes removal of chylomicron remnants, intermediate density lipoprotein particles (IDL), and the highly atherogenic low-density lipoprotein particles (LDL) from circulation. The process is mediated by certain receptors on the hepatocytes and controlled by the function of several apolipoproteins including apoB, apoE, and ApoC-III. Therefore, liver function is a major determining factor for CVD. The underlying mechanisms of how weight loss reverses fatty liver and achieves remission of diabetes remain largely unclear [10,27,43]. Recent data from rodent studies highlighted the role of diacylglycerol (DAG) in activation of the ε isoform of hepatic protein kinase C (PKCε), which impairs insulin function, and weight loss was reported to reverse this process [44,45,46]. In addition, long-chain saturated fatty acids have also been reported to activate the toll-like receptor 4 (TLR-4) and generate toxic ceramides that inhibit insulin signaling [46,47,48].

Insulin is the master regulator of lipid metabolism; it is known to inhibit lipolysis to maintain non-esterified fatty acid (NEFA) levels by suppressing the function of hormone-sensitive lipase (HSL) [49]. Insulin is a regulator of hepatic VLDL production indirectly via downregulating transcription of the ApoC-III and the microsomal triglyceride transfer protein (MTP) [50]. It is also a regulator of the transcription factor forkhead box protein (FoxO1), which in addition to regulation of gluconeogenesis upregulates the expression of ApoC-III and MTP and thereby enhances lipidation and secretion of VLDL by the liver [51,52]. Expression of ApoC-III in the β-cell of mice impaired insulin secretion [53], whereas FoxO1 expression protected β-cell from adverse metabolic conditions [54]. Furthermore, proprotein convertase subtilisin kexin type 9 (PCSK9) may have effects in lipid metabolism and β-cell function in T2DM, and use of anti-PCSK9 monoclonal antibodies has a potential to prevent new-onset diabetes in the future, although the evidence is currently lacking [55].

Whether β-cell dysfunction in T2DM is a result of toxic lipids or related to other effector proteins produced by the β-cell itself or other organs remains an open question, and this is currently an active area of research. Hypoxia and oxidative stress are known in obesity and T2DM [56]. This new redox environment could be the initiation factor in altering lipoprotein metabolism and generation of reactive lipid species, which in turn alter adipose tissue function and the biology of β-cell to survive these lipotoxic conditions. Investigation of the biochemical changes of lipoproteins and related lipid products during development and remission of diabetes may therefore lead the way to new therapeutic targets. In this regard, proteomics and metabolomics studies using advanced mass spectrometry techniques will reveal the underlying changes in the biochemistry and function of lipoproteins and lipid-related molecules.

## 3. Lipotoxicity and β-Cell Dysfunction

Loss of β-cell function is critical factor to the pathogenesis of T2DM. Despite debate about the terminology, “lipotoxicity” remains the most widely accepted hypothesis to explain β-cell dysfunction in T2DM [16,23,24,57]. This review is directed towards giving an overview of the evidence in favor of the adverse effect of lipids on the pancreas and β-cell function, and does not cover “lipotoxicity” or “glucotoxicity” in detail. For more detailed information, other recent reviews cover the topic at cellular and molecular levels [16,23,24,58,59,60].

At normal physiological conditions, fatty acids are known to stimulate insulin secretion from the pancreatic β-cells [61,62]. Therefore, saturated fatty acids that are a major component of the modernized diet are potentially the triggers for excessive basal insulin. This in turn is permissive for a cluster of major metabolic abnormalities including insulin resistance, NAFLD, and dyslipidemia [59]. Various hypotheses to explain the glyco-lipotoxic effects on β-cell function in T2DM have been postulated, including apoptosis, endoplasmic reticulum (ER) stress, oxidative stress, inflammation, mitochondrial dysfunction, autophagy, and de-differentiation [16,22,23,63,64]. However, the precise mechanisms of how toxic lipids can induce stress and eventually dysfunction of the β-cell remain critically important to be established [7,22].

Most of the available data on “lipotoxicity” are based on in vitro studies of β-cell lines or isolated islets, and most confirmed the deleterious effects of palmitate in causing ER stress or apoptosis when incubated with β-cells at high concentration [65,66]. Incubation of β-cells with palmitic acid (C16:0) combined with either oleic acid (C18:1) or arachidonic acid (C20:4) prevented the cellular damage that was induced by palmitic acid alone [65,67,68]. On the other hand, incubating β-cells with arachidonic acid enhanced β-cell proliferation and increased insulin secretion in cultured cell lines and β-cells [67,68]. It is important to consider that all these in vitro studies used a high concentration of fatty acids that are not encountered under physiological conditions, and hence there is no concrete in vivo evidence yet of the proposed lipotoxic effect of fatty acids in humans [57]. Under physiological conditions, β-cells are exposed to a mixture of nutrients including glucose, fatty acids, and amino acids, and thus the term “nutri-stress” that was proposed recently by Prentki et al. is more appropriate instead of “lipotoxicity” or “glucolipotoxicity” [23].

Fatty acids have been reported to contribute to the β-cell dysfunction on the basis of the number of carbons in the fatty acyl chain and the degree of saturation. Saturated fatty acids with long chain (i.e., C16:0 palmitic acid) have been reported to induce cell death or apoptosis whereas unsaturated fatty acids (i.e., C18:1 oleic acid) had opposing effects [59,60,68,69]. In the support of these cellular findings, it has been demonstrated that palmitate-induced ER stress was modulated by the expression of stearoyl-CoA desaturase and Elovl6 in rodents [70,71].

In vivo work on β-cell lipotoxicity was pioneered over 30 years ago by Roger H. Unger in rodents [18,72]. In the ZDF rat, triglyceride content of the islets increased 10 times during development of T2DM, a few weeks before development of hyperglycemia, and this correlated strongly with circulating fatty acids. In addition, this rise in islet fat associated with absence of glucose-stimulated insulin secretion and low expression of GLUT-2 of the β-cells [18]. Using the same model, it was found that infusion of intralipid and glucose decreased β-cell function in older but not in younger animals [73]. However, it is important to consider that intralipid mainly contains unsaturated fatty acids that were reported not to have toxic effects on β-cells.

The evidence of lipotoxic effect of fatty acids is less significant in humans, although there is a general agreement about the synergic effects of glucose and fatty acids on β-cell dysfunction in T2DM [23,57,74]. “Metabolic inflexibility” of the cell to switch between glucose and fatty acid metabolism is well known [75], and this is supported by our recent indirect calorimetry data showing a decrease in lipid oxidation accompanied by an increase in glucose oxidation after remission of T2DM [27]. It was reported recently that palmitic acid is not effective fuel for β-cell in rodents [76,77], and this may partially explain β-cell dysfunction in T2DM. Some observational and lipid infusion studies have reported association between NEFA and β-cell function in humans, whereas others found no such evidence [23,57,78,79,80]. Interestingly, using positron emission tomography (PET), researchers found that fatty acid uptake by the pancreas was higher in obese individuals compared with normal weight controls, and this was associated with lowered glucose uptake and blood flow, which was associated negatively with markers of β-cell function [81].

It is important to recognize that the regional level of NEFA is under tight control of insulin, and this is normally regulated by cross talk between the liver, adipose tissues, and pancreas [49,82]. In addition, circulating NEFA makes only one portion of the fatty acids encountered by the pancreas, and the concentration of NEFA in blood is low [83]. There are several other sources for β-cell uptake of fatty acids. (I) Lipoprotein lipase (LPL) and hence uptake from circulating triglyceride can regulate β-cell function [84]. (II) Adipocyte infiltration in close proximity to the pancreatic islets in T2DM and hydrolysis of triglyceride content are other sources of fatty acids for the β-cells [65,85]. (III) Formation of lipid droplet within the β-cell was found in T2DM [86], and high expression of HSL in β-cell has decreased insulin secretion after feeding mice on a high fat diet, and this was associated with lower triglyceride accumulation within the islets of the transgenic mice compared with the wild-type mice [87]. Furthermore, toxic lipid intermediates derived from fatty acid metabolism including diacylglycerides and ceramides were confirmed to cause damage to insulin signaling within the hepatocytes and myocytes [88,89], but less is known about how such toxic metabolites can affect β-cells [7,57]. Thus, the lack of correlation between plasma NEFA and β-cell dysfunction does not provide evidence against the lipotoxic effect of lipids on β-cell function.

Earlier studies claimed that apoptosis could explain loss in β-cell mass and function in T2DM [64,90,91,92]. However, there is no strong evidence to support β-cell death from either animal or human studies [23,30,93]. On the other hand, loss of specialized β-cell phenotype (de-differentiation) could be explained by glucolipotoxicity [63,94,95,96,97], and this is the most likely mechanism to explain return of β-cell function after remission of T2DM [10,98]. Under metabolic conditions of excess fat and eventually glucose, some β-cells lose their identity to become glucagon-producing α-cells [96,99]. Conclusive data confirming β-cell de-differentiation are limited, especially in humans [63,96]. More work is needed to confirm whether de-differentiation/re-differentiation are the main underlying molecular mechanisms of β-cell dysfunction and recovery in T2DM.

## 4. The Link between Lipoprotein Export and β-Cell Dysfunction

Is there clear evidence of the link between lipoprotein export and “lipotoxicity” or dysfunction of the β-cell? The “Twin Cycle” hypothesis postulates that hepatic lipoprotein export is the upstream pathway that delivers excess fat to the pancreas and leads eventually to β-cell dysfunction [28]. However, until now, there has been no direct evidence to support the idea that VLDL-TG export is the source of fat buildup within the pancreas, and this remains a hypothesis (Figure 1). Indirectly, we have shown that remission of diabetes is associated with fall in hepatic VLDL-TG export, and return to diabetes status has been associated with increase in plasma VLDL-TG levels [43]. Direct uptake of VLDL particles by the β-cells was reported in humans, and expression of LPL within the β-cell was found in mice and affected insulin secretion [84,100]. Isolated islets from mice lacking the LDL receptor showed lower LDL uptake and more survival rate than mice that expressed the LDL receptor [101]. In addition, it was reported that LPL is expressed in the capillaries of pancreatic islets, and this ensures delivery of postprandial fatty acids from chylomicrons to the β-cells [83]. Local expression of ApoC-III within the pancreatic islets has led to β-cell failure in mice [53]. In contrast, it was found that high-density lipoprotein (HDL) expression in β-cell had a protective role against ER stress [102].

High intrapancreatic fat content and infiltration of adipocytes in close proximity to pancreatic islets are known in T2DM [65,103]. Recently, we have demonstrated that normalization in liver fat content and hepatic VLD-TG export is related to the fall in the palmitic acid component of VLDL-TG, the pathway that delivers fat to the peripheral tissues including the pancreas [43]. This fatty acid is the obligatory product of DNL and the most toxic fatty acid to the β-cell at high concentration and under prolonged exposure [69]. Interestingly, loss of β-cell function during relapse into diabetes was found to be associated with a rise in plasma levels of VLDL-TG, highly enriched with palmitic acid, and an increase in intrapancreatic fat levels (Figure 2).

Despite evidence about the role of toxic lipids on the pathogenesis of NAFLD, there are limited data on such effects on the β-cells in humans [57]. This is mainly due to the limited access to human pancreatic tissues. Unlike the liver, pancreas biopsy is a very invasive process and is not feasible in clinical practice. Available data in humans are derived from pancreas samples collected from post-mortem donated organs and excised tissues during surgery, which are not ideal conditions and could partially explain the conflicting reports [104]. There is urgency to develop non-invasive imaging techniques to study β-cells in vivo because of these obstacles, and novel PET-MRI methods using safe tracers are currently under development [105].

It has been known for a long time that the biology and structure of pancreatic islets are different in human and rodents, and this is reflected in the β-cell’s ability for adaptation to adverse metabolic conditions [106,107,108]. Therefore, recent work should focus on human studies considering these major differences between species. Recent RNA-sequencing data from human islets showed that saturated fatty acids induced β-cell stress independent of the major inflammatory pathways [109]. A recent study reported that higher expression of the cluster differentiation 36 (CD36) receptor is associated with defective insulin secretion in β-cells from obese donors with T2DM [110]. CD36 is a transporter protein that determines fatty acid uptake by the β-cells and could be a potential target to block β-cell uptake of these toxic lipid products. In addition, this CD36 receptor was found to be localized on the insulin granules in human β-cells and found to control fatty acid stimulation of insulin secretion [111]. Further work is required to identify potential toxic lipid species and the underlying mechanisms of their toxic effects on β-cell function to inform alternative treatments for T2DM.

## 5. Adipokines and Lipid Related Markers

Lipid homeostasis in humans is maintained by high coordination between the liver, the pancreas, and the adipose tissues. Although it has been considered for long time as a storage depot, the metabolic function of adipose tissues has gained more interest in recent years after the discovery of leptin and adiponectin [112]. Adipose tissue function should therefore be taken into consideration when discussing β-cell dysfunction, T2DM, and CVD.

Leptin is predominantly produced by adipocytes, and plasma levels reflect total body fat mass; thus, leptin level is always higher in women than men at any given BMI [113,114]. Leptin regulates fat storage as well as appetite and has been associated with both glucose- and lipid-lowering effects [18,19,20]. Adiponectin, another adipocyte-derived hormone, has an antidiabetic effect [21,22]. In contrast to leptin, adiponectin level is negatively correlated with adiposity [23]. The plasma leptin to adiponectin ratio is considered a marker of atherogenicity in T2DM [115,116]. It is known that leptin level is elevated in obesity and T2DM, and resistance to leptin action is widely accepted [117,118]. Dietary weight loss has been reported to cause decrease in leptin and increase in adiponectin levels [119,120,121,122].

Inflammation in adipose tissue is likely a consequence of excess fat accumulation. However, the role of inflammatory cytokines on adipose tissue biology is uncertain. Whether or not inflammatory cytokines contribute to β-cell dysfunction and CVD development requires further investigation, and this is beyond the scope of this review [123,124]. Low-grade inflammation was found to be associated with C16:0 ceramide, and lowering plasma levels of this lipid species was reported as a potential therapy for T2DM [125,126]. However, saturated fatty acids were found to induce ER stress in β-cells independent of the major inflammatory pathways [30,31].

It has been suggested that lipids and branched-chain amino acids (BCAAs) could have a synergic effect in causing insulin resistance in T2DM, but little is known about their effect on β-cell function [127]. Several studies have reported elevated plasma BCAAs in obesity and T2DM [128,129]. In rodents, BCAAs were reported to stimulate insulin secretion and activate the mTORC1, which is related to β-cell mass and function [130]. mTORC1 is a negative regulator of autophagy [131], and calorie restriction modulates autophagy, a process that known to regulate lipid metabolism [132,133,134,135]. There are conflicting reports as to whether the change in BCAA level after weight loss in human is related to weight loss per se or to diabetes status [134,135].

The growth and differentiation factor 15 (GDF-15) is related to nutritional stress, and it is elevated in T2DM and CVD [136,137]. In addition, the fibroblast growth factor 21 (FGF-21) is secreted by the liver and is highly expressed in the pancreas. FGF-21 is strongly associated with obesity and T2DM as a regulator of lipid and glucose metabolism [138,139,140]. It selectively promotes glucose uptake in adipocytes, and thereby complements the function of leptin and adiponectin in promoting TG storage in adipocyte and fatty acid oxidation in other tissues [140,141,142]. FGF-21 levels are elevated in patients with T2DM, possibly suggesting resistance to its function [117,138,143]. In rodents, FGF-21 stimulates adiponectin, which exhibits ceramidase activity, degrading toxic ceramides and enhancing insulin sensitivity [144,145,146]. In addition, hepatic expression of FGF-21 was reported to enhance the cellular process that leads to browning of adipose tissues [147,148]. Notably, brown adipose tissues, which contribute to regulation of body metabolism and energy expenditure via oxidation of glucose and lipids, are more abundant in women [149]. Furthermore, it has been shown that FGF-21 shifts VLDL-TG uptake from white to brown adipose tissues in obesity [150]. Interestingly, FGF-21 was found to regulate hepatic VLDL-TG secretion in mice via accelerating adipose tissues uptake mediated by CD36 and LPL functions [150].

Identification of generic biomarkers for cell stress and differentiation would be useful to understand the underlying mechanism of β-cell failure and recovery in T2DM in light of the lack of β-cell-specific dysfunction markers [136,138,139]. Further work is needed to understand the exact functions of GDF-15, FGF-21, and BCAAs in relation to lipid metabolism and β-cell function in humans.

## 6. Intrapancretic Fat and Pancreas Morphology

Although the pancreas is the most important organ for diabetes, studies on it are limited. Clinical and observational studies of the pancreas have naturally focused on islet function itself, but the relevance of acinar cells to endocrine function has rarely been considered [151,152,153,154]. The pancreas volume is small in T2DM, with marked irregularity in pancreas borders [155,156]. Acinar cell mass reflects total pancreas volume with islet and ductal systems contributing ≈5%. In addition, fat content of the pancreas is moderately elevated in T2DM [25,27,157], correlating negatively with pancreas volume [155]. Recently, we have confirmed that normalization of intrapancreatic fat content returned β-cell function with normalization of maximal β-cell capacity [158].

Studies on post-mortem pancreas of people with T2DM have shown fibrosis in exocrine tissues associated with decline in β-cell and increase in α-cell mass [159]. In ZDF rat, fat replacement of the acinar cells developed into fibrosis [160], possibly leading to destruction of the islets and β-cell dysfunction [153,160,161,162]. Furthermore, trans-differentiation of the acinar cells into adipocytes occurs in mice during ageing, regulated through expression of c-Myc transcription factor [163]. Whether these reported changes in acinar cells are related or secondary to loss of β-cell function is an important question. Inadequate insulin secretion during development of T2DM could possibly explain the observed atrophy of pancreas volume due to lack of trophic effect of insulin on pancreatic tissue [164]. Notably, pancreas volume is small in people with type 1 diabetes (T1DM) when insulin secretion is absent [165,166]. The trophic effects of insulin at high concentrations such as experienced by pancreatic tissues following a meal could be considerable [164,167]. Insulin-like growth factor 1 (IGF-1) level is low in T1DM, ageing, and T2DM [168,169,170,171]. IGF-1 receptor shares high homology with insulin receptor, and cross-reactivity of insulin with IGF-1 receptor bring about tissue growth effects at a high concentration of insulin. It is possible that the lack of both IGF-1 and insulin are involved in the loss of acinar cell mass in T2DM. Further work on the underlying molecular mechanisms, particularly the role of IGF-1 and c-Myc, is required to understand the relevance of pancreas morphology to the pathogenesis of T2DM.

**Figure 3:**
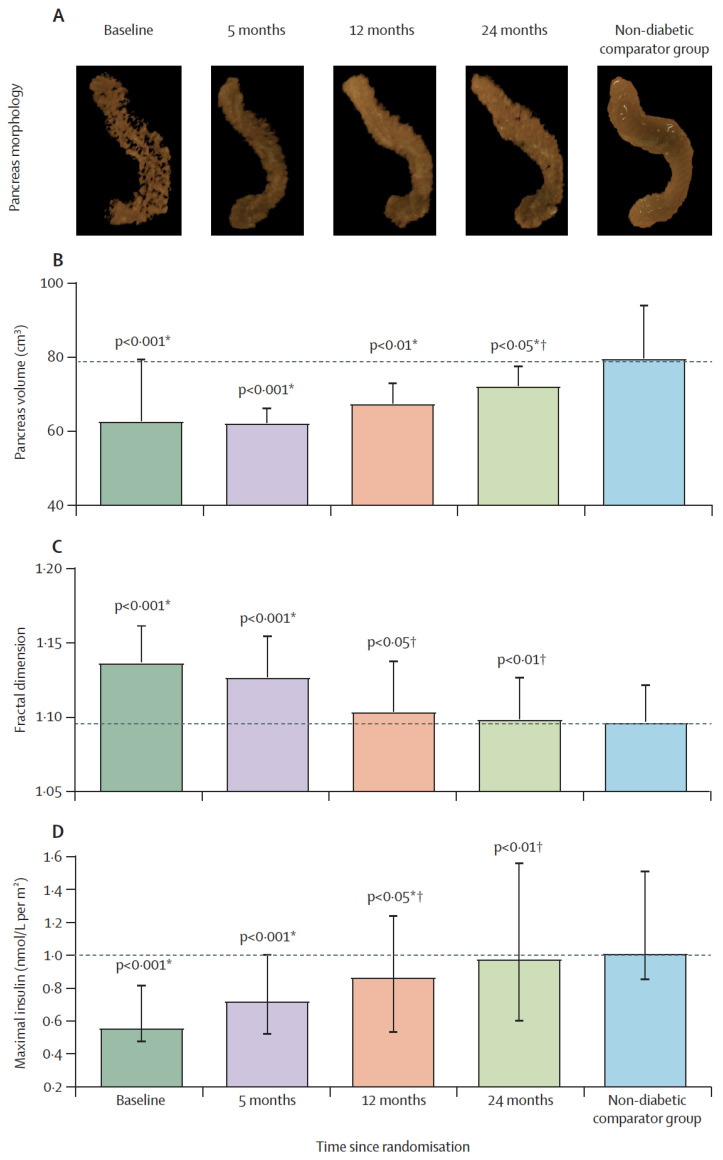
Restoration of pancreas morphology and β-cell functional capacity after 2 years of remission of type 2 diabetes. Surface-rendered image of pancreas morphology in a representative responder (**A**), and pancreas volume (**B**), fractal dimension (**C**), and maximal insulin secretion (**D**) in responders compared with non-diabetic controls at baseline, 5 months, 12 months, and 24 months. Horizontal dashed lines indicate the level for non-diabetic controls. Data paired with baseline at each timepoint are presented as mean (SD) for pancreas volume and fractal dimension and median (IQR) for insulin secretion. * Responders versus non-diabetic comparator group. † Responders versus baseline. Figure is presented with permission from The Lancet Diabetes and Endocrinology [172].

It has been reported that lipid droplets are more common within acinar cells of human donors with T2DM [86], and higher expression of the CD36 fatty acid transporter protein was associated with defective insulin secretion [39]. Recently, we have demonstrated that remission of T2DM and recovery of β-cell function were associated with gradual normalization of the pancreas morphology (Figure 3) [172]. The increase in pancreas volume after removal of excess fat suggests that this could be mediated by high post-meal insulin surges and other factors. Regeneration of pancreas tissues is known, and this may explain the observed increase in pancreas volume during remission of diabetes [173]. Whether the underlying mechanisms of toxic effect of fatty acids are similar between β-cells and acinar cells remains to be elucidated in human. Ultimately, this could lead the way to novel strategies to maintain β-cell function and preserve normal pancreas health.

## 7. Lipoprotein Metabolism and CVD Risk in T2DM

People with T2DM face a considerably increased risk of CVD due to disordered lipoprotein metabolism [32,174,175]. The risk of atherosclerosis has historically been attributed to the rise in hepatic secretion of triglyceride-rich VLDL, decrease in HDL cholesterol, and the rise in the small, dense LDL particles [175]. However, ApoB has been considered a more useful factor in determining CVD risk than the traditionally used LDL cholesterol levels [176]. The risk of incident T2DM has been reported to be determined by the size of the lipoprotein particles rather than lipid content and hence by the dynamics of lipoprotein secretion and clearance by the liver [177]. There is currently increased interest in the effect of lipoprotein (a), which is found to be a risk factor for CVD [178,179].

Apolipoproteins (ApoB/ApoE) are important for the assembly and clearance of lipoproteins [50,180,181]. Lack of clearance of lipoproteins remnants was reported to be related to interference with the binding of ApoB/ApoE receptors via high hepatic expression of ApoC-III [182,183]. However, it was reported recently that ApoC-III promotes LPL activity independent of ApoE-mediated clearance of lipoproteins [184]. There are conflicting reports as to whether plasma level of ApoE can predict CVD [185,186]. However, it should be recognized that not all the LDL particles in circulation carry ApoE, and thus ApoE level does not necessarily reflects the atherogenic nature of the whole lipoprotein population. Polymorphisms of ApoE gene and ApoC-III were reported to increase the risk of developing T2DM and CVD, which requires further investigation [181,187]. In addition, high expression of ApoC-III within the pancreatic islets caused β-cell failure in mice [53]. Downregulation of ApoC-III expression could therefore be used as a therapeutic target to enhance lipolysis, decrease β-cell damage, and prevent CVD risk.

Women have a lower risk of T2DM and CVD than men [13,14]. However, once T2DM develops, the risk rises to a very high level in men [188,189]. Plasma level of VLDL-TG is higher in men than in women [190], pointing to the difference in hepatic lipoprotein export and clearance. It is likely that adequate subcutaneous fat storage capacity may permit safe clearance of liver exported fat in women. However, once these stores become inflamed or reach maximal capacity to safely store excess fat, women are especially prone to excess lipid damage. Higher levels of leptin and adiponectin in women are consistent with the protective nature of these markers in developing T2DM and CVD [17,113,149].

The recent guidelines of the European Association for the Study of Diabetes (EASD) and the European Society of Cardiology (ESC) recommend statins for lowering lipid profile and prevent CVD mortality in patients with T2DM in both sexes, although the potential differences between women and men were highlighted [191].

## 8. Mechanisms of Remission of T2DM after Weight Loss

Weight loss studies have highlighted the likely deleterious effect of excess fat on β-cell function [25,26,27]. In T2DM, hepatic lipoprotein export is associated with high intrapancreatic fat accumulation and loss of β-cell function [26,27,43]. Decrease in both hepatic and intrapancreatic fat is a prerequisite for diabetes remission with ultimate dependence on β-cell ability to recover function after removal of this metabolic stress [27,43]. Furthermore, weight regain and loss of remission have been found to be associated with a major rise in hepatic VLDL-TG production and plasma VLDL-TG concentration (Figure 2). These data are consistent with the “Twin Cycle” hypothesis that was proposed to explain the etiology and reversibility of T2DM [28,192]. This specific enrichment of palmitic acid, known to be toxic to the β-cells, within the lipoprotein exported from the liver is consistent with a causative effect on β-cell dysfunction. The degree of weight loss, lipoprotein export, and weight stability were the major predictors for remission [43,193]. Other studies that investigate the molecular mechanisms of long-term body weight maintenance after weight loss are required. RNA sequencing of adipose and skeletal muscle biopsies showed that acylcarnitine species can determine weight regain after weight loss [194]. This result is consistent with our recent finding of the rise of palmitic acids within the VLDL-TG during weight regain and loss of remission [43], and also in agreement with the regulatory function of CerS6-dependent C16:0 ceramide on weight gain in mice [47]. Collectively, these data highlight novel approaches for the treatment of obesity and T2DM in the future.

Bariatric surgery has long been known to be effective in normalizing blood glucose and achieving remission of T2DM [195]. We have shown that the underlying physiology of diabetes reversal after surgery is the same as dietary weight loss and related to removal of ectopic fat from the liver and the pancreas [157]. The general similarity of mechanisms has also been reported by others [196,197]. Although minor improvement in β-cell function happen within a week after surgery, long-term normalization requires a longer time to remove excess fat from the pancreas [157,198]. The increase in post-meal levels of glucagon-like peptide-1 (GLP-1) immediately after surgery is unlikely to contribute to the observed rapid normalization of fasting plasma glucose. It is likely to be related to surgery procedures themselves, given that GLP-1 levels do not change after dietary weight loss despite similar glycemic improvement [119,199]. In contrast, the level of ghrelin increases following dietary weight loss but not after bariatric surgery [119,193,195]. Taken together, neither GLP-1 nor ghrelin causes remission of diabetes following weight loss, and the rise in the levels of these hormones reflects counter-regulatory mechanisms to the effects of surgery and hunger, respectively.

Long-term nutrient overload leads to loss of β-cell specialized function in secretion insulin, and removal of the lipotoxic environment will decrease uptake of the toxic lipids and restore β-cell function [22,28]. The demonstrated return of β-cell function after removing the metabolic stress following weight loss is not compatible with β-cell death or “apoptosis” [10,24,98]. Regeneration of β-cell is another process that may account for recovery β-cell mass after remission, and some drugs have been reported to have such proliferative effects. However, evidence of β-cell replication or proliferation is lacking in adult humans [108,200,201].

An appropriate diet is critical for DNL regulation and metabolic health. A diet that contains a high level of carbohydrates is known to increase DNL rate, whereas a ketogenic diet has opposing effects [202,203]. A low-calorie diet that restricts methionine was shown to delay diabetes development in mice [204]. In addition, BCAA restriction improves metabolic health in both mice and humans [205]. Given the insulin secretion stimulatory effect of BCAAs on β-cells [130], the high levels of BCAAs observed in obesity and T2DM may suggest a deleterious long-term effect or resistance to their function similar to what happens to FGF-21 in obesity [128,129,138]. Restriction of BCAAs was reported recently to worsen ketogenesis and cause oxidative dysfunction of the mitochondria in mice [206]. A ketogenic diet could be a useful approach to burn excess calories and harness less energy by the cell theoretically, although randomized controlled trials have shown only a minor effect [207]. However, more studies should be carried out to assess the adverse metabolic effects of this extreme diet including high-circulating NEFA and their potential lipotoxic effects [203].

The β-cell workload hypothesis has also been proposed to explain dysfunction and decline in β-cell mass in T2DM [64,208]. This is essentially based on the high demand on β-cells to increase insulin secretion in obesity, leading eventually to β-cell death or dysfunction. The hypothesis also explains the ethnicity difference between Asians and Caucasians in terms of the discrepancy in β-cell capacity to increase mass during obesity and T2DM. Recent reports on remission of diabetes and return of β-cell function following weight loss fits well with the workload hypothesis if discussed in the context of “lipotoxicity” and dysfunction of β-cell rather than death [10,158]. Calorie restriction not only lifts the chronic nutrient burden encountered by the β-cells, but also will remove the stimulus to produce excess circulating insulin. This is critical in triggering the cellular processes that favor use of energy rather than storage via downregulation of lipogenesis genes and normalization of hepatic lipid metabolism. Further studies to understand the exact mechanisms of β-cell recovery after weight loss are critically important for sustained remission of T2DM.

## 9. Novel Directions to Study Pancreas and Lipid-Mediated β-Cell Dysfunction

At normal physiological conditions, palmitic acid stimulates β-cells in secreting insulin [61,62,209]. However, prolonged exposure of β-cells to this stimulatory effect will cause stress, which is the trigger of lipotoxicity or β-cell dysfunction [23,59]. It is possible that chronic stimulation of β-cells indirectly leads to β-cell dysfunction and development of CVD by altering hepatic lipoprotein metabolism and the biology of adipose tissue. Therefore, healthy β-cell function is central for prevention of T2DM and maintaining normal cardiovascular health [7]. Current pharmacological therapies such as sulfonylureas that increase insulin secretion are potentially harmful to β-cells in the long-term. Future therapies should be targeted to remove the lipotoxic environment and hence to support β-cells to return to normal function.

At diagnosis of T2DM, the majority of β-cells (60–70%) have already lost function. Although the process takes almost a decade, there is currently no method to non-invasively assess the progressive deterioration of the pancreas during development of T2DM prior to rise of blood glucose levels [105,210]. Our work has shown that dysfunction of β-cell and decline of pancreas volume are associated with high levels of intrapancreatic fat and increase in diabetes duration [26,155]. It would be reasonable to postulate that the abnormal pancreas morphology in T2DM is driven by the same mechanism that causes β-cell dysfunction (“lipotoxicity”), and hepatic DNL is a likely initiating factor [43]. The DNL pathway could be modulated at early stage during progression of T2DM.

Although it is widely accepted, the “lipotoxicity” hypothesis to explain β-cell dysfunction is not yet proven, especially in human studies [57]. Further research is required to identify the potential toxic lipid species or combinations and to determine how exactly they induce cellular dysfunction. Access to human pancreatic tissues is critical, especially when advanced imaging techniques are utilized [211]. Advanced imaging mass spectrometry (IMS) techniques on precision cut pancreatic slices would be very useful for in situ localization of lipid species within the pancreatic tissues in close proximity to the islets. In this regard, the Time-of-Flight Secondary Ion Mass Spectrometry (TOF-SIMS) system could be useful in getting a full profile of lipid species at subcellular resolution [212,213]. It could be used to determine the chemical structure of lipids within the lipid droplets inside the adipocytes, and within individual β-cells or acinar cells. MALDI (TOF/TOF) imaging could also be used for co-localization of lipid binding protein receptors (CD36) [214]. IMS data could be confirmed by high-resolution electron and confocal microscopy imaging. Flow cytometry imaging, metabolimetry, and live cell imaging are other complementary techniques that could also be useful [215,216,217]. In addition, lineage tracing studies could be illuminating for identification the origin of β-cells, α-cells, and adipocytes within the pancreatic tissues [218].

Excessive expansion of adipose tissues changes the biology of adipocytes, decreasing their secretory ability of adipokines. This unhealthy situation will activate pro-inflammatory macrophages that produce inflammatory cytokines including tumor necrosis factor alpha (TNFα) and interleukin 6 (IL-6) [219]. Non-invasive imaging techniques that can detect early signs of adipose tissue inflammation could be useful in assessing the capacity of fat storage as a risk factor for developing T2DM and CVD. In addition, understanding the molecular basis of adipose tissue expansion is critical for modeling their function. The peroxisome proliferator-activated receptor-γ (PPARγ) is a transcriptional regulator of adipocyte maturation and an essential factor mediating the function of these cytokines and regulation of insulin secretion [220,221]. Perilipin 5 (PLIN5), which is another transcriptional regulator of adipocyte, was found to regulate insulin secretion in the islets of humans and mice in a cAMP-dependent manner, and to prevent the lipotoxic damage to β-cells [222,223]. It is possible that excessive insulin secretion leads indirectly to β-cell dysfunction and eventfully development of CVD by altering hepatic lipoprotein metabolism and promoting unhealthy expansion of adipose tissues.

**Figure 4:**
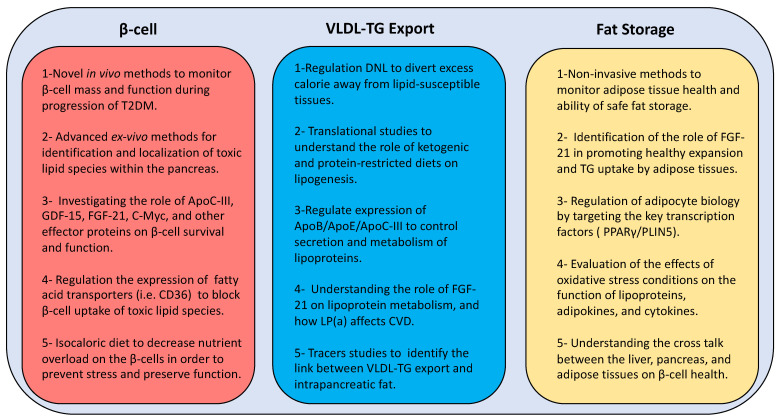
New directions of research for developing novel therapies to manage type 2 diabetes. T2DM: type 2 diabetes mellitus; GDF-15: growth differentiation factor 15; FGF-21: fibroblast growth factor 21; C-myc: cellular myelocytomatosis oncogene; β-cell: beta cell; CD36: cluster differentiation 36; DNL: de novo lipogenesis; ApoB: apolipoprotein B; ApoE: apolipoprotein E; ApoC-III: apolipoprotein C-III; LP(a): lipoprotein (a); VLDL-TG: very low density lipoprotein triglyceride; PPARγ: peroxisome proliferator-activated receptor-γ; PLIN5: perilipin 5.

A combination of human and animal studies is required to understand the toxicity of lipid species on β-cell function and the role of the above transcription factors on lipid metabolism and β-cell survival. Ultimately, this could lead the way to novel strategies (Figure 4) for prevention and remission of T2DM other than the current blunt weight loss approach. Early actions are required to monitor pancreas health through the development of novel in vivo imaging techniques. In addition, localization of toxic lipid species is essential to understand the underlying mechanisms via developing advanced ex vivo imaging methods at cellular and subcellular levels. Furthermore, identification of the potential oxidative post-translational modifications of lipoprotein and lipid-related moieties during development of T2DM will open new windows for advanced targeted therapies in the future.

## 10. Conclusions

The pancreas is a key regulator of hepatic lipids, and healthy pancreas function is central for regulation of whole-body metabolism. The decline in acinar cell mass and loss of β-cell function are two major pathological process associated with excess intrapancreatic fat in T2DM. Therefore, early non-invasive diagnosis methods are useful for maintaining pancreas health and prevention of T2DM and related cardiovascular complications.

The classical definition of “lipotoxicity” should not be restricted to the deleterious effects of fatty acids. It should be extended to include the role of other lipid species including lipid-related markers and signaling molecules. Despite indirect evidence of the lipotoxic effects on β-cell function in T2DM, more work is required to identify the nature and function of the potential toxic lipid species. Advanced in situ imaging will be invaluable to identify and colocalize lipid species at subcellular resolution. This will inform developing novel therapeutic strategies via use of neutralizing antibodies or ligands that can decrease or regulate cellular uptake of these toxic products. For example, regulation of the expression of the CD36 transporter would be a promising approach in the future to block β-cell uptake of palmitic acid and related toxic intermediates.

Novel therapeutic strategies for remission of T2DM should focus on the key pathophysiological pathways that are regulated after weight loss. Effective strategies should initially decrease excess insulin and alter the lipotoxic environment. Use of insulin secretion enhancing drugs increases the metabolic burden on remaining β-cells, possibly leading to non-reversible damage. Notably, hepatic lipoprotein export pathway should be targeted to allow safe storage of excess calories. This could be achieved by dietary or pharmaceutical means via targeting the key regulatory elements (i.e., DNL, ApoC-III, LPL). Genetic, cellular, and translational studies accompanied by advanced in vivo imaging and mass spectrometry techniques will increase our knowledge in the future about the mechanisms behind unhealthy expansion of adipose tissues and β-cell dysfunction. This should be exploited to design novel strategies to promote storage of fat away from ectopic sites and decrease cellular exposure to toxic lipids. In this regard, FGF-21 has great effects in regulation the uptake of excess triglycerides and selective expansion of adipose tissues, and could potentially lead to attractive therapeutic options.

## Data Availability

Not applicable.
